# Hand trauma in English domestic professional county cricket

**DOI:** 10.17159/2078-516X/2021/v33i1a10689

**Published:** 2021-08-03

**Authors:** WJ Ribbans, MS Chaudhry, B Goudriaan

**Affiliations:** 1 Northamptonshire County Cricket Club, Northampton, England; 2 The University of Northampton, Northampton, England; 3 The County Clinic, 57 Billing Road, Northampton, England NN1 5DB

**Keywords:** digits, fractures, dislocations, injury

## Abstract

**Background:**

Hand trauma is a frequent and disabling injury in cricket. However, there is limited published data on its impact on the sport at the elite level.

**Objectives:**

This study investigated the incidence and mechanism of hand injuries in professional cricket over a decade and the impact of these injuries upon player availability.

**Methods:**

A retrospective hand injury review at Northampton County Cricket Club (NCCC) over 10 years (2009–2018) was performed. All hand injuries had been contemporaneously documented. They were analysed for cause of injury, treatment, and time away from competitive play.

**Results:**

There were 45 hand injuries in total. Eleven percent needed surgical intervention. These hand injuries required a total recovery time of 1561 days, and in-season 1416 days were lost from competitive play. The injuries requiring surgery were unavailable for 229 total days during the season. A player had an annual 18% risk of sustaining a hand injury requiring time away from the sport and resulting in a 4% reduction in playing resources during a season.

**Conclusion:**

Hand injuries have major implications for player selection during the cricket season and place a potential burden upon the entire squad and the team’s success.

The incidence of injury in cricket has been reported to be increasing.^[1,2]^ Lower limb injuries have been recorded at between 23–50% of total injuries.^[3,4]^ The upper limb accounts for between 20–34% overall, with hand and wrist injuries most strongly represented.^[1,3,5,6,7]^ Hand injuries mainly affect the digits as fractures and dislocations.

Hand trauma can occur during any of the main activities – batting, bowling, and fielding (including wicket-keeping). However, fielding has been repeatedly reported to be where most of the injuries occur-mainly via direct impact with the ball or ground.^[1,2,8,9,10]^ Injuries can occur pre-season and during the season, during matches and training.

The aim of this study was to investigate the incidence and mechanism of hand injuries in a professional cricket club over a decade and to assess the impact of these injuries on the availability of playing squad members.

## Methods

Injuries to players at the Northamptonshire County Cricket Club (NCCC) were recorded on a computerised injury surveillance programme to which the players had consented. Subsequently this data was available for analysis and auditing. The period of study was from 2009–2018 (ten seasons). Two of the authors (WR and BG) were affiliated to the NCCC throughout the study period.

The competitive season for cricket lasts between the months of April and September. During this time, the club is involved in three competitive formats – the County Championship (four day games), 50-over 1-day games, and 20/20 competitions. In addition, there are pre-season games, matches against touring countries, and second XI games.

Hand injuries were defined as any injury distal to the base of the metacarpals for the purpose of this analysis. The injuries included osseous, articular, and soft tissue damage. Imaging was available for bone and joint injuries. The number of players available to the senior squad was recorded. Although some players may feature predominantly (or exclusively) in one or two of the formats only, each individual was recorded as being available throughout the entirety of the season.

The date of return-to-play (RTP) was used for defining injury recovery - although it is acknowledged that a period of graduated training would need to be undertaken beforehand.

If an injury occurred pre-season or the recovery continued after the season ended, the time the player was unavailable for selection during the season, as well as the total length of recovery, was measured. Players requiring out-of-season rehabilitation for injuries remained at the club for treatment with one of the authors (BG). This allowed an assessment of the timing of full cricket-related recovery.

## Results

Forty-five injuries to twenty-two different players were recorded over the ten-year period of study. None were recurring injuries.

### Type and site of injury

**Fig. 1:**
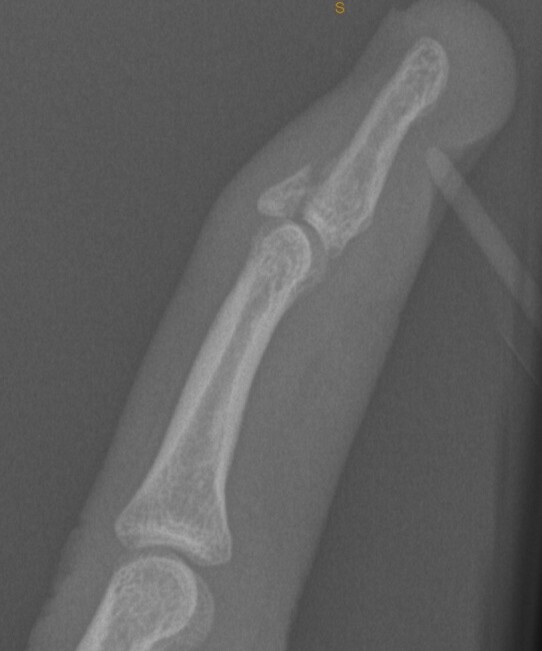
Lateral x-ray of ring finger**:** large dorsal avulsion fragment at insertion of Extensor digitorum longus (EDL) following catching injury

Purely non-articular fractures contributed to 31 (69%) of the total hand injuries and articular injuries (including dislocations, fracture-dislocations, tendon avulsions and ligament injuries for 13 (29%) of the injuries (Fig. 1). The final injury was a nail bed haematoma which required time away from the game.

The left hand was involved in 53% of injuries and the right hand in 47%. The little finger accounted for 24% of all injuries, ring finger 31%, middle 11%, index 9%, and thumb 24%. The metacarpals, including the metacarpophalangeal joints (MCPJs), were involved in 11% of injuries. Table 1 provides analysis of the precise site of the injuries. Although not this paper’s area of study, it should be noted that no wrist or carpal injuries were recorded over the study period.

### Treatment

Five injuries (11%) required surgical intervention. The remainder of the injuries were treated conservatively.

### Timing and activities involved in injuries

Thirty-two (71%) of the injuries occurred during matches and 13 (29%) during practice sessions of which four (31%) occurred preseason.

Fifty-six percent of injuries occurred during fielding activities, 20% specifically while wicket-keeping, and 24% during batting. No injuries occurred during bowling.

### Recovery from injuries

The 45 injuries resulted in a total of 1561 days off for rehabilitation. During the season, a total of 1416 days lost. On average, a hand injury led to 31 days/player (range 0–89) time lost for selection for games. Five injuries required surgery and the players were unavailable for 229 days during the ten seasons, resulting in an average of 46 days/player time lost for selection. Conservatively treated injuries resulted in 1187 days off during the season which averaged 30 days/player time lost for selection.

Most English professional cricket seasons last an average of 22 weeks. Therefore, a club might expect to endure 92 % of the season with playing selection reduced because of a hand injury. During the ten-year period, Northamptonshire had an average of 25 senior players available for selection across three formats. Hand injuries reduced this capacity by 4%.

## Discussion

Cricket is a popular global sport. The International Cricket Council (ICC) has over 100 member countries affiliated to it.^[11]^ Whilst played as a non-contact sport between participants, injuries do occur and are common. Impact injuries frequently result from contact between the hard ball and body parts. Ball speeds during bowling can be greater than 160 km.h^−1^.^[11]^ In addition, impact injuries occur from direct contact with the ground, another player, or the pitch boundaries.^[12]^

Injuries at both professional and recreational levels impact upon individual player performance and team success. However, the incidence of cricket injuries has been reported to be lower than in other common team sports^[1,8]^ - although injury rates have varied greatly in different publications. Weightman recorded 2.6 injuries/10000 hours played ^[13]^, whilst Orchard reported 24.2 injuries/10 000 hours played ^[1]^ – a tenfold difference. Leary and White calculated an acute injury rate of 57.4/1 000 days of cricket played.^[14]^ Hammond recorded a match incidence of 3.2 injuries/1 000 hours exposure compared to 29.9 in soccer and 97.6 in rugby union.^[9]^ Injury surveillance reports indicate that a return to cricketing activities from all injuries occur in one week or less in 47.8 %, 28.4% within two to three weeks, and 23.8% over more than three weeks.^[2]^ The differences in recorded rates may reflect the increasing sophistication of injury surveillance methods over time, the criteria used for recording an injury, and whether the publication concentrated upon one sport or involved ‘across sport’ comparisons with varying fixture scheduling and physical demands.

This study demonstrates that hand injuries sustained in professional cricket have significant implications for player welfare, availability, and selection. These results constitute the longest continuous period of analysis (ten years) of any study on hand injuries in elite cricket. It represents the experience of the equivalent of 250 years exposure to playing the sport professionally. Additionally, it calculates how long it takes to recover before play is resumed again and the impact on senior player availability during a professional season.

A professional cricketer had an 18% annual risk of sustaining a hand fracture over the course of this study. Whilst only 11% of the injuries required surgical intervention, the time away from the sport still averaged seven weeks. This emphasised the importance of hand comfort, strength, and flexibility to perform all of the necessary skills within the game. At any one time, coaching staff, managing an average squad of 25 players, could expect to lose throughout the season an average of one player to these injuries alone.

Our data indicate that both hands are at equal risk and the digits more prone to injury than the metacarpals and MCPJs. Our study confirms the results from previous smaller studies that the ring and little finger are most at risk - combining to cater for 56% of all injuries.^[8]^ Fielding activities were the most common cause of injury, similar to the findings in previous literature.^[8],[15]^ Batting and wicket-keeping activities are afforded some hand protection through the use of gloves but they are not immune to injury. The absence of any wrist or carpal injuries during the decade of analysis in this paper emphasises the burden the game places specifically on the digits.

Brooks’ ^[16]^ recent paper reports an Australian experience of 90 hand fractures identified over a three-year period across many elite teams. Their surgical intervention rate of 13% is similar to that in our study (11%). Ninety-three percent of their fractures involved the thumb, 5^th^ ray, and middle and distal phalanges of the index, middle and ring fingers – the ‘exterior bones’. This was comparable to our study with 89% of hand fractures. The return-to-play statistics between the two studies is also similar. Conservatively treated injuries averaged 30 days in our study (32 days in Australia) and 46 days for surgical cases (49 days in Australia).

‘Buddy strapping’ the 4^th^ and 5^th^ digits has previously been advocated to reduce injury during fielding practices ^[10]^ and would seem to be a reasonable precaution but without any proven data to support it. Specific gloves for catching practice are available but provide better protection to the palm and metacarpals than the digits. It is acknowledged that players want to replicate the skill of catching during practice and compliance for the use of more constrained gloves might be low.

An acknowledged limitation of this study includes the precise mechanism of fielding injuries - whether they occurred via direct impact with the ball, contact with the cricket field, or other methods of injury. Although the data recorded the format of game for match injuries, the sample size was too small for meaningful analysis of differential injury patterns in other competitions.

## Conclusion

Hand injuries have major implications for player selection during the cricket season. Coaching staff should be aware of the potential burden such injuries place upon the entire squad and the impact for a team’s sporting success. An evaluation of fielding techniques, practice drills, and future new protective equipment might contribute to a decrease in such injuries.

## Figures and Tables

**Table 1:** Anatomical location, treatment, timing, and cause of hand injuries

	Thumb	Index	Middle	Ring	Little	Total
**Total number of injuries**	**11 (24%)**	**4 (9%)**	**5 (11%)**	**14 (31%)**	**11 (24%)**	**45**

**Anatomical Location**						
Distal phalanx	5	2	5	7	3	22 (49%)
Distal Interphalangeal Joint (including mallet finger injuries)	0	0	0	1	4	5 (11%)
Middle phalanx	0	1	0	2	1	4 (9%)
Proximal Interphalangeal Joint (including ligamentous injury)	1	0	0	1	1	3 (7%)
Proximal phalanx	3	0	0	0	2	5 (11%)
Metacarpophalangeal joint (including ligamentous injury)	0	0	0	0	0	0
Metacarpal injuries	2	0	0	3	0	5 (11%)
Soft tissue injuries (excluding above)	0	1	0	0	0	1 (2%)
**Treatment**						
Conservative treatment	11	3	4	11	11	40 (89%)
Surgical treatment	0	1	1	3	0	5 (11%)
**Timing of injuries**						
Matches	9	1	4	8	10	32 (71%)
Training	2	3	1	6	1	13 (29%)
**Activity at time of injury**						
Batting	3	3	1	2	2	11 (24%)
Bowling	0	0	0	0	0	0
Fielding (excluding wicket-keeping)	5	1	2	11	6	25 (56%)
Wicket-keeping	3	0	1	1	4	9 (20%)
**Total time lost during season post-injury (days)**	378	83	144	536	275	1 416
**Total time lost post-injury (days)**	421	140	144	561	295	1 561

Data are expressed as n or n(%), where % indicates the percentage of the total number of injuries.
